# Supplementary material for: Direct Mass Spectrometry-Based Detection and Antibody Sequencing of Monoclonal Gammopathy of Undetermined Significance from Patient Serum: A Case Study
Source: J Proteome Res. 2023 Jul 27;22(9):3022–8. doi: 10.1021/acs.jproteome.3c00330 (PMC10476240; doi:10.1021/acs.jproteome.3c00330)
Supplement: Supplementary file 1 — pr3c00330_si_001.pdf [file pr3c00330_si_001.pdf]

Supporting Information to:

Direct mass spectrometry-based detection and antibody sequencing of Monoclonal Gammopathy of Undetermined Significance from patient serum – a case study.

Weiwei Peng[1], Maurits A. den Boer[1], Sem Tamara[1], Nadia J. Mokiem[1], Sjors P.A. van der Lans[2], Douwe Schulte[1], Albert Bondt[1], Pieter-Jan Haas[2], Monique C. Minnema[3], Suzan H.M. Rooijakkers[2], Arjan D. van Zuilen[4], Albert J.R. Heck[1], Joost Snijder[1*]

[1] Biomolecular Mass Spectrometry and Proteomics, Bijvoet Center for Biomolecular Research and Utrecht Institute of Pharmaceutical Sciences, Utrecht University, Padualaan 8, 3584, CH, Utrecht, The Netherlands

[2] Medical Microbiology, University Medical Center Utrecht, Utrecht University, Heidelberglaan 100, 3584CX Utrecht, The Netherlands

[3] Department of Hematology, University Medical Center Utrecht, Utrecht University, Heidelberglaan 100, 3584CX Utrecht, the Netherlands

[4] Department of Nephrology and Hypertension, University Medical Center Utrecht, Utrecht University, Heidelberglaan 100, 3584CX Utrecht, the Netherlands

* corresponding author: j.snijder@uu.nl

Contents:

- Supplementary Table S1. Intact mass analysis of MGUS Fab and derivatives.
- Supplementary Figure S1. Longitudinal IgG1 Fab profile of the MGUS patient.
- Supplementary Figure S2. Tandem MS spectrum of N-glycopeptide in CDRH1 of M-protein.
- Supplementary Figure S3. Middle-down LC-MS/MS spectra of M-protein Fd and LC.

Supplementary Table S1. Intact mass analysis of MGUS Fab and derivatives. Glycoforms are denoted with: N-acetyl hexosamine (N), hexose (H), fucose (F), and N-acetyl neuraminic acid (S).

Sample	glycoform	mass exp. (Da)	SD (Da)[c]	mass theor. (Da)[d]	Δmass (Da)	relative intensity (%)
intact Fab[a]	N5H5F1	50093.96	0.40	50090.79	3.17	3.63
	N4H5F1S1	50181.62	0.16	50178.85	2.77	7.52
	N5H5F1S1	50384.96	0.21	50382.05	2.91	22.04
	N4H5F1S2	50472.65	0.02	50470.11	2.54	23.78
	N5H6F1S1	50549.69	0.04	50544.19	5.50	4.93
	N5H5F1S2	50675.96	0.13	50673.30	2.66	32.57
	N5H6F1S2	50837.75	0.17	50835.45	2.30	5.54
Fab + PNGase F[a]	-	48120.43	0.12	48117.67	2.76	-
Fab + TCEP HC[b]	N5H5F1S2	26952.69	0.37	26951.09	1.60	-
Fab + TCEP LC[b]	-	23729.78	0.37	23731.31	-1.53	-

[a] *native MS*

[b] *LC-MS*

[c] *average and standard deviations are calculated across the series of charge states for native MS, across 4 replicate measurements for LC-MS.*

[d] *average theoretical mass considering disulfide bond formation and pyroglytamic acid conversion of the heavy chain N-terminus.*

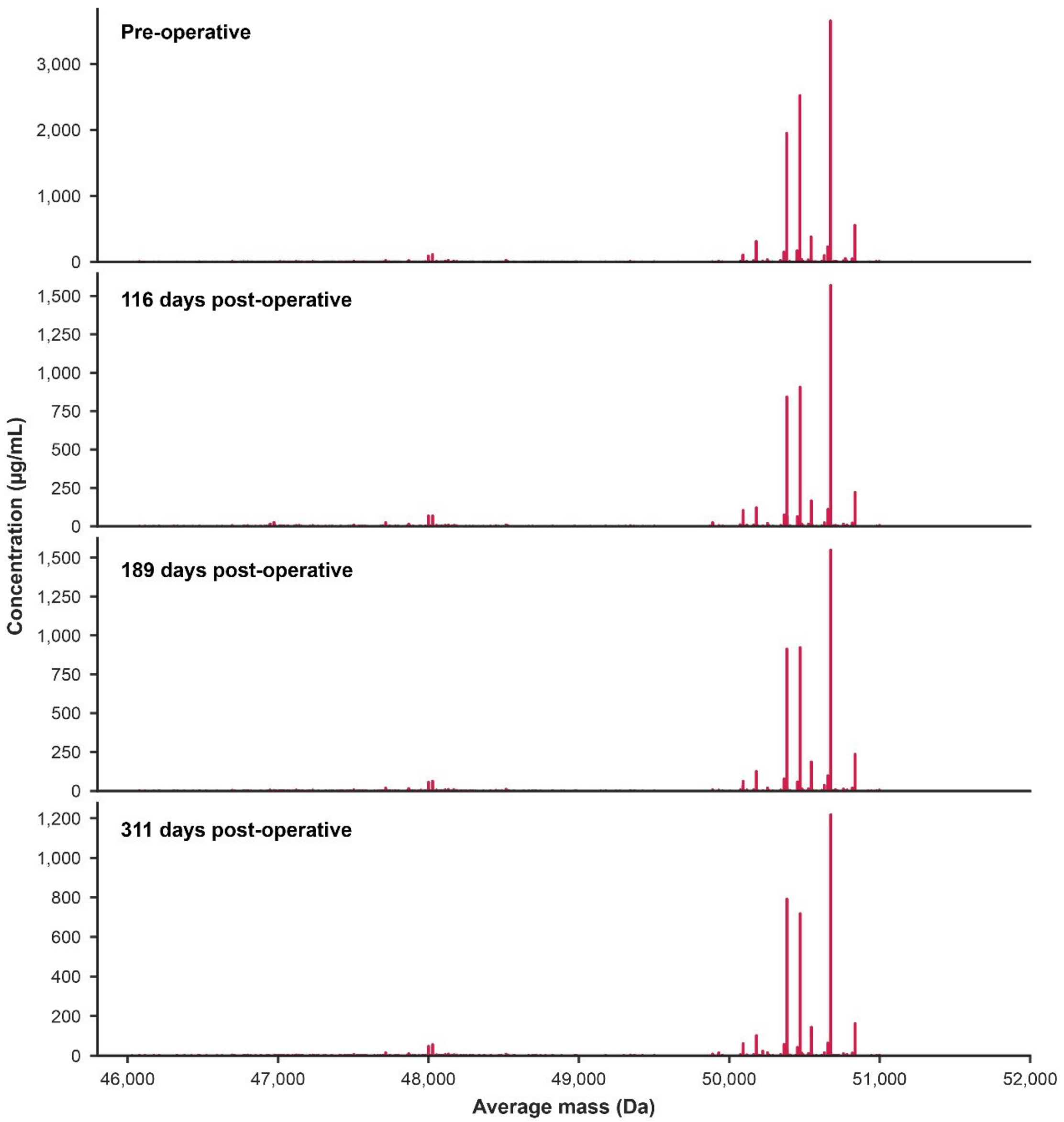

Supplementary Figure S1. Longitudinal IgG1 Fab profile of the MGUS patient.

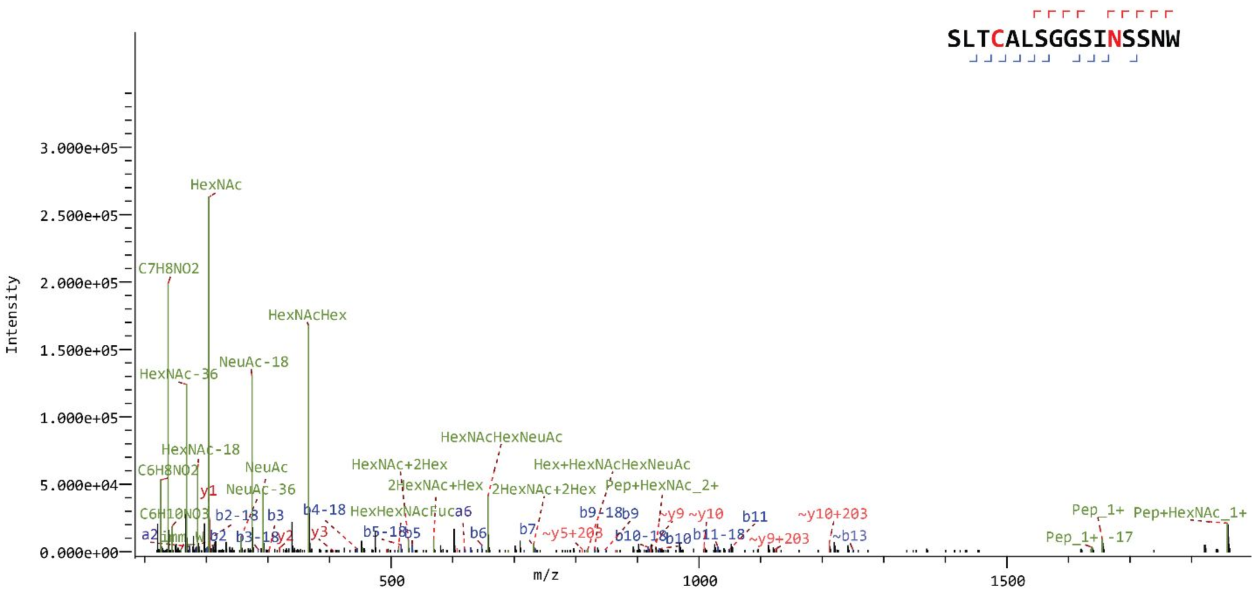

Supplementary Figure S2. Tandem MS spectrum of N-glycopeptide in CDRH1 of M-protein. Shown is the HexNAc(5)Hex(5)Fuc(1)NeuAc(2) glycoform.

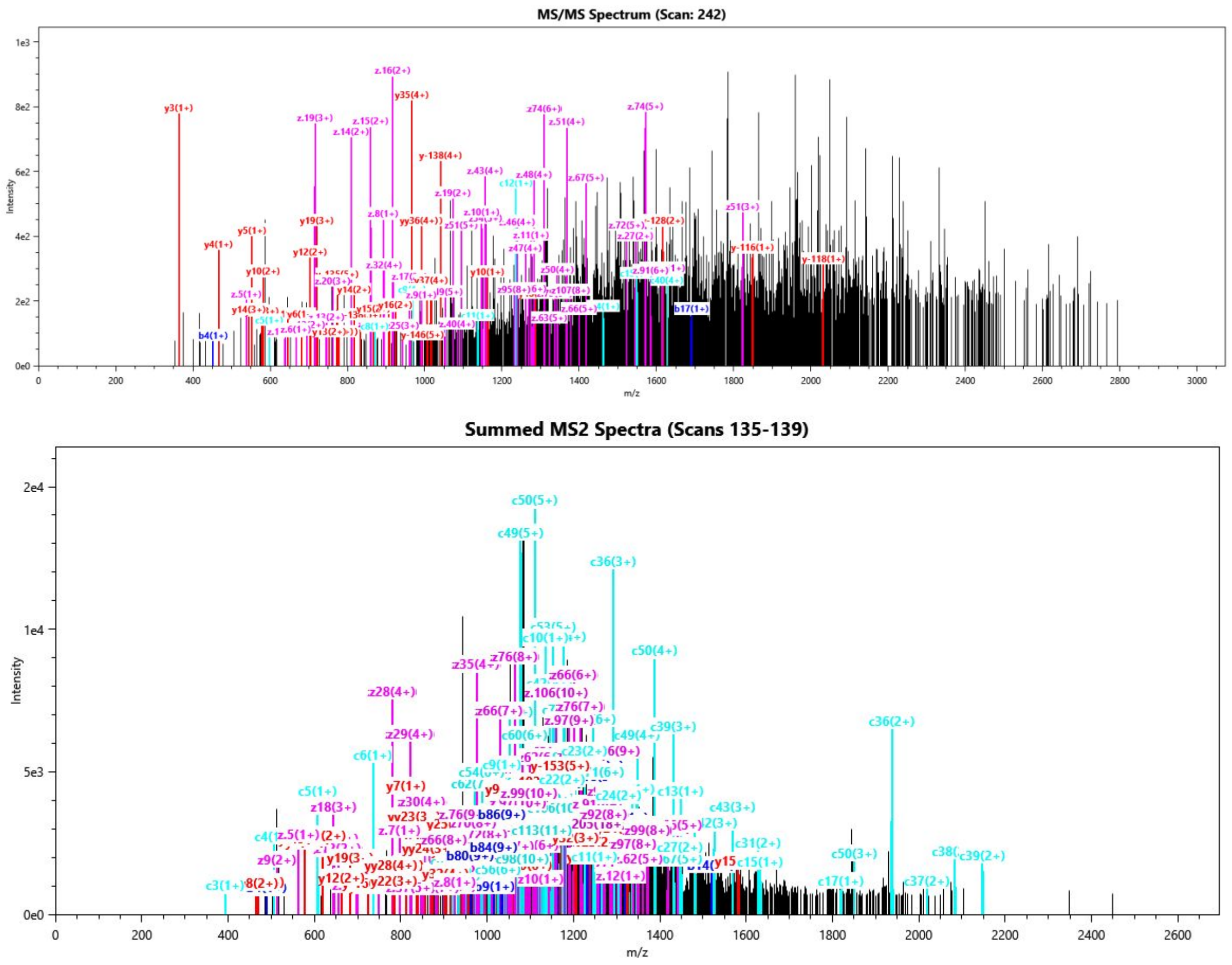

Supplementary Figure S3. Top-down LC-MS/MS spectra of M-protein Fab Heavy Chain (top) and Light Chain.
